# Right Ventricular Free Wall Strain and Congestive Hepatopathy in Patients with Acute Worsening of Chronic Heart Failure: A CATSTAT-HF Echo Substudy

**DOI:** 10.3390/jcm9051317

**Published:** 2020-05-02

**Authors:** Josip A. Borovac, Duska Glavas, Zora Susilovic Grabovac, Daniela Supe Domic, Lada Stanisic, Domenico D’Amario, Darko Duplancic, Josko Bozic

**Affiliations:** 1 Department of Pathophysiology, University of Split School of Medicine, Soltanska 2, 21000 Split, Croatia; jborovac@mefst.hr; 2 Institute of Emergency Medicine of Split-Dalmatia County, Spinciceva 1, 21000 Split, Croatia; 3 Clinic for Cardiovascular Diseases, University Hospital of Split, Spinciceva 1, 21000 Split, Croatia; duska.glavas@gmail.com (D.G.); zoragrabovac@gmail.com (Z.S.G.); dduplanc@mefst.hr (D.D.); 4 Department of Internal Medicine, University of Split School of Medicine, Soltanska 2, 21000 Split, Croatia; 5 Department of Medical Laboratory Diagnostics, University Hospital of Split, Spinciceva 1, 21000 Split, Croatia; daniela.supedomic@gmail.com (D.S.D.); lada.stanisic@gmail.com (L.S.); 6 Department of Health Studies, University of Split, Rudjera Boskovica 35 P.P. 464, 21000 Split, Croatia; 7 Department of Cardiovascular and Thoracic Sciences, IRCCS Fondazione Policlinico A. Gemelli, Università Cattolica Sacro Cuore, Largo Francesco Vito 1, 00168 Rome, Italy; domenico.damario@gmail.com

**Keywords:** echocardiography, heart failure, heart failure decompensation, hepatic insufficiency, laboratory markers, liver dysfunction, ventricular dysfunction, right

## Abstract

Right ventricular (RV) function is an important predictor of prognosis in patients with heart failure. However, the relationship of the RV free wall longitudinal strain (RV FWS) and the degree of hepatic dysfunction during the acute worsening of heart failure (AWHF) is unknown. We sought to determine associations of RV FWS with laboratory liver function tests and parameters of RV function including tricuspid annular plane systolic excursion (TAPSE), RV fractional area change (RV FAC), maximal tricuspid jet velocity (TR V_max_), RV S′ velocity, and estimated RV systolic pressure (RVSP). A total of 42 AWHF patients from the CATSTAT-HF study were stratified in two groups by the RV FWS median (−16.5%). Patients < RV FWS median had significantly prolonged international normalized ratio (INR; *p* = 0.002), increased total bilirubin (*p* < 0.001) and alkaline phosphatase (ALP; *p* = 0.020), and decreased albumin (*p* = 0.005) and thrombocytes (*p* = 0.017) compared to patients > RV FWS median. RV FWS independently correlated to total bilirubin (*β* = 0.457, *p* = 0.004), ALP (*β* = 0.556, *p* = 0.002), INR (*β* = 0.392, *p* = 0.022), albumin (*β* = −0.437, *p* = 0.013), and thrombocytes (*β* = −404, *p* = 0.038). Similarly, TAPSE, RV FAC, and RV S′ significantly correlated with RV FWS. In conclusion, RV impairment, reflected in reduced RV FWS, is independently associated with a higher degree of hepatic dysfunction among patients with AWHF (CATSTAT-HF ClinicalTrials gov number, NCT03389386).

## 1. Introduction

The ability of right ventricle (RV) to maintain forward cardiac output and prevent systemic venous congestion in the setting of left-sided heart failure (HF) due to systolic and/or diastolic dysfunction becomes an essential determinant of clinical outcomes [1]. Previous studies have shown that RV dysfunction is associated with poor prognosis and reduced exercise capacity among patients with both ischemic and dilated cardiomyopathy and reduced left ventricular ejection fraction-LVEF (HFrEF) [2,3,4,5]. Similarly, RV dysfunction and pulmonary artery hypertension (PAH) are highly prevalent among patients with HF and preserved ejection fraction (HFpEF) and are both associated with worse clinical sequelae and advanced syndrome severity [6,7,8]. RV dysfunction in HFpEF is caused by both RV contractile impairment and afterload mismatch from PAH and is independently associated with elevated pulmonary artery pressures, presence of atrial fibrillation, male sex, and LV failure [7].

RV dysfunction and HF, in general, are commonly associated with impaired hepatic function while HF and liver disease often coexist [9]. Cardiohepatic interactions are complex and bidirectional since cardiac dysfunction can induce cardiogenic liver injury; right-sided HF might lead to congestive hepatopathy, while liver cirrhosis, nonalcoholic fatty liver disease, and liver transplantation can exacerbate and complicate cardiac dysfunction [9]. Both RV failure and biventricular failure give rise to central venous hypertension leading to necrosis in the central zone of hepatic lobules, the release of inflammatory cytokines, and hepatic cholestasis resulting in hepatic hypoxia, parenchymal atrophy, necrosis, collagen deposition, and ultimately, fibrosis [10]. Consequently, laboratory liver function tests (LFTs) are commonly abnormal in the setting of acute and chronic HF or during the worsening episode of chronic HF, in the absence of primary significant liver disease or acute hepatic failure, and these derangements are associated with poor outcomes [11,12,13].

On the other hand, RV free wall longitudinal strain (RV FWS) has recently emerged as a speckle-tracking echocardiographic method providing a feasible and reproducible measure of RV systolic function and its mechanics [14]. RV has a complex morphology due to its noncylindrical form and exhibits different hemodynamic qualities compared to LV [1]. Since there is a predominance of longitudinal and oblique myofibers in the RV free wall and the fact that interventricular septum is shared by both ventricles, it is considered that RV FWS better corresponds to RV mechanical function, in sensu stricto, than RV global strain or similar RV indices [15]. In line with this, RV FWS has been identified as the potent predictor of cardiovascular and all-cause mortality among patients with HFrEF [16,17,18] and PAH [19], independently of LV systolic function and traditional parameters of RV function.

Since RV dysfunction in HF has been implicated in hepatic derangements, we hypothesized that impaired RV systolic function, as reflected in the reduced RV FWS, would adversely affect biochemical parameters of liver function. Of note, the relationship of RV FWS with hepatic function in general and also among patients with HF has been unexplored thus far. Therefore, in the present study, we sought to measure RV FWS, as the indicator of RV function, among patients with acute worsening of chronic HF (AWHF) and in the absence of significant primary liver disease or acute liver injury. Our main objective was to determine the potential association of RV FWS with laboratory LFTs in this patient cohort. Of secondary importance was to determine the association of RV FWS with traditional parameters of RV function and estimated pulmonary artery pressures.

## 2. Materials and Methods

### 2.1. Study Population and Legal Information

We consecutively included a total of 42 patients (1:1 ratio, 21 women and 21 men) in the study of which all have completed advanced deformation imaging via speckle-tracking echocardiography including the analysis of 2D right ventricular free wall strain (2D RV FWS) and left ventricular global longitudinal strain (LV GLS) along with standard transthoracic echocardiography (TTE) examination. Patients were recruited from the original CATSTAT-HF study of which baseline patient characteristics, study design, and inclusion/exclusion criteria are described elsewhere in detail [20]. A study took place at the Clinic for Cardiovascular Diseases of University Hospital of Split, Croatia between January 2018 and February 2019. All patients provided informed written consent. The study was conducted according to the principles laid out in the Declaration of Helsinki, whereas Ethics Committee of the University Hospital of Split (approval number 2181-147-01/06/M.S.-17-2) and the Ethics Committee of University of Split School of Medicine approved the study protocol. Finally, the study was preregistered at the ClinicalTrials.gov registry before the enrollment of the first patient (January 3, 2019) under the acronym CATSTAT-HF (Serum Catestatin Expression and Cardiometabolic Parameters in Patients with Congestive Heart Failure—ClinicalTrials number: NCT03389386).

### 2.2. Study Inclusion and Exclusion Criteria

In short, in this echo substudy, we consecutively enrolled patients of both sexes with acute worsening (decompensation) of chronic HF that were in New York Heart Association (NYHA) functional class II–IV. All patients had a previously verified and documented history of HF. None of the patients had NT-proBNP levels < 300 pg/mL (“rule-out” criteria) and all included patients fulfilled age-adjusted NT-proBNP criteria (“rule-in” criteria) upon admission, according to established studies [21,22]. Diagnosis of the acute worsening episode of HF in terms of physical signs and symptoms was adjudicated by the on-site investigator that was a licensed cardiologist with HF subspeciality, and this was carried out according to the current European Society of Cardiology (ESC) guidelines for the diagnosis and treatment of acute and chronic HF [23]. None of the enrolled patients had hepatomegaly upon clinical examination and documented history of primary significant liver disease, acute liver injury, nonalcoholic steatohepatitis (NASH), cholestasis, history of alcohol abuse, an autoimmune disorder, primary pulmonary disorder, or significant hematological abnormalities. None of the patients had de novo HF, active infectious disease, active malignant disease, or acute coronary syndrome/stroke as the underlying etiology of their current presentation.

### 2.3. Patient Assessment and Diagnostic Work-Up

Patient demographic, anthropometric, and clinical characteristics were recorded at admission.

Ischemic cardiomyopathy was defined as the documented previous history of myocardial infarction (defined as non-ST segment myocardial infarction-NSTEMI or ST-elevation myocardial infarction-STEMI and verified by the medical records). All patients underwent physical examination, 12-lead electrocardiogram (ECG), and chest X-ray imaging as per our standard institutional protocol for patients with dyspnea. Furthermore, all patients enrolled in the study received a standard TTE examination and advanced myocardial deformation analysis within 24 h of hospitalization, while imaging data was stored on the local workstation. Respective strain analyses were conducted “off-line” from the obtained images, when appropriate and as detailed in Section 2.4.

Furthermore, all patients had their peripheral blood sampled from the antecubital vein within 24 h of admission and the morning of the next day after fasting for laboratory analyses. All laboratory analyses were undertaken in the same certified in-hospital laboratory and handled by the same specialist in medical biochemistry that was an investigator in the present study. Liver function tests and cholestasis markers were obtained from all patients, and data were included on aspartate aminotransferase (AST), alanine aminotransferase (ALT), alkaline phosphatase (ALP), gamma-glutamyltransferase (GGT), total bilirubin, and albumin. A basic coagulation panel was ordered for every patient and consisted of prothrombin time (PT) normalized to the international normalized ratio (INR), activated partial thromboplastin time (APTT), and D-dimer assay. The N-terminal pro-B-type natriuretic peptide (NT-proBNP) levels were determined at the first contact by using Elecsys^®^ Cobas e601 NT-proBNP assay and electrochemiluminescence (ECLIA) method (Roche Diagnostics GmbH, Mannheim, Germany), high-sensitivity cardiac troponin I (hs-cTnI) was measured on the Abbott Architect ci16200 analyzer (Abbott Diagnostics, Chicago, IL, USA), while catestatin and soluble suppression of tumorigenicity (sST2) serum levels were determined by the enzyme-linked immunosorbent assay (ELISA) by using commercially available diagnostic kits (EK-053-27CE, EIA kit for CST and EK-036-10 kit for sST2, Phoenix Pharmaceuticals Inc., Burlingame, CA, USA). The C-reactive protein (CRP) was measured by immunoturbidimetry method on the Abbot Architect ci16200 platform. Other laboratory parameters were determined by using standard laboratory methods according to good laboratory practice. Fatty Liver Index was calculated by the online calculator by incorporating data obtained from body mass index (BMI, kg/m^2^), waist circumference (cm), GGT (IU/L), and triglycerides (mmol/L) [24]. The estimated glomerular filtration rate (eGFR) was calculated by the Chronic Kidney Disease Epidemiology Collaboration (CKD-EPI) formula [25].

### 2.4. Echocardiography

A comprehensive TTE examination was performed in all patients on the same day when blood samples were collected. All images were acquired using Vivid^TM^ 9 ultrasound system (GE Medical Systems, Milwaukee, WI, USA), stored digitally, and analyzed off-line on the Echo PAC workstation installed on the PC (EchoPac PC, version 112; GE Medical Systems, Milwaukee, WI, USA). Data were acquired from parasternal long- and short-axis views and three standard apical views while for each view, cine loops were acquired by recording three consecutive cardiac cycles. All patients referred for echocardiography had a verified structural and/or functional myocardial alteration in conjunction with symptoms and fulfilled NT-proBNP criteria for HF. A single on-site investigator who was a cardiology consultant with expertise in cardiac ultrasonography and clinical experience in speckle tracking methods (Z.S.G.) performed all echo analyses including speckle-tracking deformation studies. A standard set of TTE measurements were taken while patients were at rest and in the left lateral decubitus position and all echocardiographic methods adhered to recommendations for cardiac chamber quantification by echocardiography in adults were endorsed by the American Society of Echocardiography (ASE) and the European Association of Cardiovascular Imaging (EACVI) [15]. LVEF was measured by the two-dimensional (2D) biplane method according to modified Simpson’s rule, and the average value of multiple measurements was recorded [26].

Left ventricular global longitudinal strain (LV GLS) is an advanced echocardiographic index reflecting longitudinal shortening of LV during systole and can detect early subclinical myocardial dysfunction before LVEF is impaired [27]. LV GLS was measured from three LV apical views (long-axis, four-chamber, and two-chamber) by using 2D speckle tracking at a frame rate of 60–80 frames/s and a semiautomatic algorithm (calculated by the EchoPac software). Aortic valve closure was identified on the 2D image. The region of interest (ROI) was calculated automatically and, where needed, adjusted manually to account for the thickness of the LV myocardium. Accordingly, LV was divided into 17 segments, while LV GLS was calculated automatically as the mean value of the global peak systolic strains acquired from each of the three views. LV GLS is expressed as a negative number with negative values closer to zero reflecting worse LV GLS, while the more negative numbers are considered to indicate better LV GLS.

RV function was examined according to the European Society of Echocardiography guidelines for the echocardiographic assessment of the right heart in adults [28]. This examination included the measurement of tricuspid annular plane systolic excursion (TAPSE, mm), RV fractional area change (RV FAC, %), lateral tricuspid annular peak systolic velocity (RV S′, cm/s), and the maximal tricuspid regurgitation jet velocity (TR V_max_, m/s). TAPSE was acquired from the apical four-chamber (4-CH) view by placing an M-mode cursor through the tricuspid annulus and measuring the amount of longitudinal motion of the annulus at peak systole. For the measurement of RV FAC, RV end-diastolic area (RVED area) and RV end-systolic area (RVES area) were measured by manual planimetry, and RV FAC was calculated by the formula RV FAC = ((RVED area − RVES area)/RVED area) × 100. The RV S′ velocities were acquired by pulse-wave tissue Doppler in the apical 4-CH view and by placing sample volume (2–4 mm) in the middle of the basal segment of the RV free wall. Peak S′ velocities were measured in 5 consecutive cardiac cycles and the average value of these measurements was recorded. The TR V_max_ was assessed by the continuous wave Doppler and was used to estimate RV systolic pressure (RVSP) in conjunction with right atrial (RA) pressure estimation based on the diameter and respiratory collapsibility of the inferior vena cava. RVSP was, therefore, calculated by using the simplified Bernoulli equation: RVSP = (4(V)^2^ + estimated RA pressure), where V represents TR V_max_ (m/s). In some of the cases in which tricuspid regurgitant jets were not of sufficient quality, pulmonary artery pressure was estimated from the pulmonary flow acceleration time.

A two-dimensional speckle-tracking echocardiography of the RV free wall consisted of acquiring a 4-CH view with a focus on RV, with the frame rate ranging from 60 to 80 frames/s. A loop consisting of at least three cardiac cycles was recorded with good visualization of the RV lateral free wall to have at least one complete cardiac cycle (usually the middle one) for the analysis without truncation. Gain settings were manually optimized for the best visualization of the RV free wall. ECG gating was ensured in all patients in order to obtain all images at nearly same heart rates. All images were acquired while patients were instructed to hold their breath in order to minimize possible breathing artifacts. RV endocardial border at the end-systolic frame was manually traced with the ROI being automatically generated by the software with further manual adjustment to include the RV wall thickness. Software analysis then automatically tracked the wall motion over a full cardiac cycle and ultrasonographer was allowed to manually readjust those segments of RV free wall that had suboptimal tracking quality. RV free wall was divided into three segments: basal, mid, and apex. The systolic longitudinal strain of the RV free wall (RV FWS) was calculated as the mean strain value recorded for basal, mid, and apical segments, while the strain of each segment was measured three times and averaged. Of note, RV FWS is expressed as a negative number where more negative numbers (higher absolute value) reflect better RV systolic function, while less negative numbers (closer to zero) reflect worse RV systolic function.

For the study purposes, the whole cohort in the present study was arbitrarily divided into two subgroups (21 vs. 21 patients) concerning the median RV FWS value that was found to be −16.5% (IQR 11.2%–20.1%) in our cohort. Accordingly, one group was designated as below median (RW FWS < 16.5%) therefore having a reduced RV FWS, while another group was designated as above median (RV FWS > 16.5%) thereby having better RV systolic function.

### 2.5. Statistical Analysis

For all statistical analyses, we used SPSS Statistics for Windows^®^ (version 26.0, IBM, Armonk, NY, USA) and Prism 6 for Windows^®^ (version 6.01, GraphPad, La Jolla, CA, USA). Data were presented as mean ± standard deviation (SD) or median (IQR-interquartile range) based on the variable distribution normality or as a number (*n*) with percentage (%). The normality of data distribution for continuous variables was assessed with the Kolmogorov–Smirnov and by visual estimation of Q–Q plots. For differences between groups, an independent samples *t*-test was employed for continuous variables with normal distribution, while the Mann–Whitney U test was used for continuous variables with non-normal distribution. Chi-squared (χ^2^) test was used to determine differences between groups with respect to categorical variables. Finally, the association of RV FWS with laboratory parameters of liver function and echocardiographic parameters of RV function were assessed through Pearson’s bivariate correlation analysis, and from this analysis, we reported correlation coefficients (r) and two-tailed statistical significance (*p*) values. To ascertain the association of RV FWS with laboratory parameters of liver function and to account for potential confounders, a multiple linear regression analysis was performed. Each variable of interest was tested in a separate regression model to reduce colinearity and potential overfitting effect, and each model was adjusted for following covariates, at all instances: age, sex, BMI, LVEF, NYHA functional class, eGFR, triglycerides, NLR, NT-proBNP levels, and estimated RVSP. From these analyses, we reported respective *p*-values and standardized *β*-coefficients with t-values. Results with the *p*-value < 0.05 were considered as statistically significant in all analyses.

## 3. Results

### 3.1. Patients’ Baseline Characteristics

All 42 patients had completed advanced echocardiographic examinations encompassing RV FWS and left ventricular global longitudinal strain (LV GLS) measurements, and this portion of the study was designated as CATSTAT-HF echo substudy. For the purpose of the analysis, patients were stratified into two groups according to RV FWS median that was −16.5% (IQR (−20.1)–(−11.2)%) in the present study, as measured. Patients in the subgroup below RV FWS median had mean RV FWS of −11.6% ± 2.8%, while those above RV FWS median had an average RV FWS of −21.3% ± 3.7%. Most of the patients were in NYHA III functional class, and the majority (*n* = 23, 54.8%) had HFrEF phenotype followed by HFpEF (*n* = 11, 26.2%) and HFmrEF (*n* = 8, 19%). More than half of patients had nonischemic cardiomyopathy and the median age was 71.5 (62–76) years. There was an equal enrollment of men and women (21 vs. 21 patients) in the echo substudy, while mean LVEF and LV GLS values were 39.1 ± 16.0% and −10.1 ± 5.5%, respectively.

Regarding comorbidities, nearly all patients had arterial hypertension (*n* = 38, 90.5%), 18 (42.9%) were former or current smokers, nearly two-thirds had dyslipidemia (*n* = 26, 61.9%), more than a third of patients had type 2 diabetes mellitus (*n* = 15, 35.7%), while 5 patients (11.9%) had concomitant chronic obstructive pulmonary disease. Furthermore, exactly half of the patients (*n* = 21) were obese (BMI ≥ 30 kg/m^2^), while almost half of the patients had atrial fibrillation (*n* = 20, 47.6%). Patients below RV FWS median were significantly older (74 (IQR 68–77) vs. 65 (IQR 54–73) years, *p* = 0.040), had significantly higher circulating NT-proBNP levels (6266 (IQR 3089–12417) vs. 2315 (IQR 1171–5590) pg/mL, *p* = 0.038), significantly higher neutrophil-to-lymphocyte ratio (5.7 ± 3.5 vs. 4.6 ± 2.9, *p* = 0.019), longer average length of stay (16.6 ± 7.8 vs. 11.7 ± 7.0 days, *p* = 0.038), and reduced percentage of lymphocytes in peripheral blood (18.8 ± 8.9 vs. 23.8 ± 6.8%, *p* = 0.047) compared to patients above RV FWS median. No significant differences were observed between two groups with respect to systolic function, LVEF and LV GLS, while they also did not significantly differ in respect to other baseline parameters (Table 1).

### 3.2. Associations of Right Ventricular Free Wall Longitudinal Strain with Laboratory Parameters of Hepatic Function

The mean heart rate of enrolled cohort at the time of echo examination was 74 ± 19 beats/min, whereas the average frame rate acquired was 68 ± 7 frames/s.

A subgroup of patients below RV FWS median had significantly higher levels of total bilirubin and ALP and significantly decreased thrombocyte count compared to patients with RV FWS above median value (Table 2). No significant differences were observed between compared subgroups in respect to AST, ALT, and GGT. Furthermore, albumin levels were significantly decreased and prothrombin time-INR was significantly prolonged among patients below RV FWS median in comparison to those above RV FWS median (Table 2).

**Figure 1:**
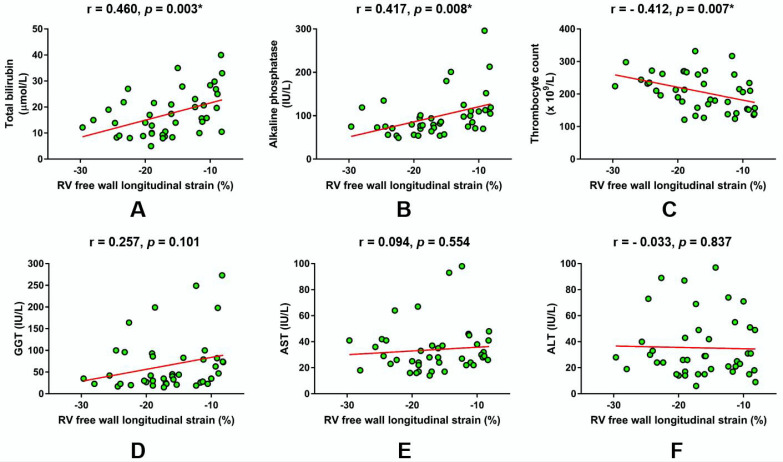
Linear regression plots showing the correlation of right ventricular (RV) free wall longitudinal strain with respect to laboratory parameters reflecting hepatic function and cholestasis. (**A**) Total bilirubin, (**B**) alkaline phosphatase, (**C**) Thrombocyte count, (**D**) gamma-glutamyl transferase, (**E**) aspartate aminotransferase, (**F**) alanine aminotransferase. * Significant statistical association at two-tailed level. r-Pearson’s correlation coefficient.

In total patient cohort (*n* = 42), a Pearson’s bivariate correlation analysis showed that RV FWS significantly correlated with total bilirubin (r = 0.460, *p* = 0.003; Figure 1A), ALP (r = 0.417, *p* = 0.008; Figure 1B), and thrombocyte count (r = -0.412, *p* = 0.007; Figure 1C). In contrast to these observations, RV FWS values were not significantly associated with GGT (r = 0.257, *p* = 0.101; Figure 1D), AST (r = 0.094, *p* = 0.554; Figure 1E), and ALT (r = -0.033, *p* = 0.837; Figure 1F) levels in the bivariate correlation analysis (Figure 1).

**Figure 2:**
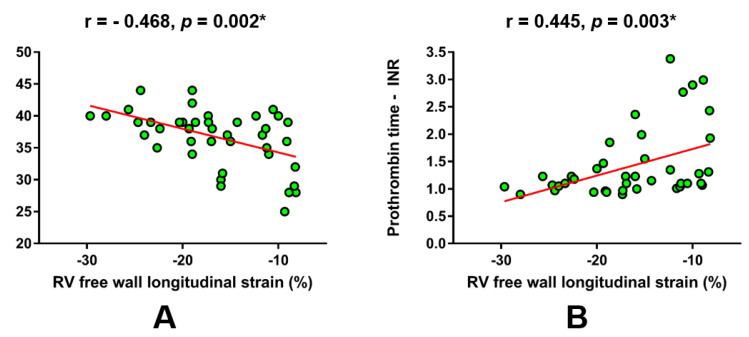
Linear regression plots showing the correlation of RV free wall longitudinal strain with respect to laboratory parameters that reflect synthetic liver function. (**A**) albumin, (**B**) prothrombin time - international normalized ratio. * Significant statistical association at two-tailed level. r-Pearson’s correlation coefficient.

Furthermore, laboratory parameters that approximate synthetic liver function were in a significant correlation with RV FWS (Figure 2). Of note, albumin levels were in a significant negative correlation with RV FWS (r = -0.468, *p* = 0.002; Figure 2A), while significant positive correlation was observed for prothrombin time-INR (r = 0.445, *p* = 0.003; Figure 2B).

When these laboratory variables were adjusted for relevant confounders in the separate models of multiple linear regression, as described in Section 2.5, it was confirmed that total bilirubin (β = 0.457, t = 3.081; *p* = 0.004), ALP (β = 0.556, t = 3.348; *p* = 0.002), albumin (β = −0.437, t = −2.629; *p* = 0.013), prothrombin time-INR (β = 0.392, t = 2.550; *p* = 0.022), and the thrombocyte count (β = −0.404, t = −2.164; *p* = 0.038) independently predicted RV FWS that was set as the dependent outcome variable. 

### 3.3. Echocardiographic Parameters of Right Ventricular Function and RV FWS

The mean values of RV function echo parameters in the whole cohort of patients were 16.3 ± 5.2 mm for TAPSE, 32.5 ± 9.5% for RV FAC, 10.0 ± 3.0 cm/s for R S′ velocity, and 2.9 ± 0.7 m/s for the TR V_max_. A subgroup of patients with RV FWS below median had significantly reduced TAPSE (13.5 ± 3.0 vs. 19.1 ± 5.4 mm, *p* < 0.001), RV FAC (28.8 ± 8.9 vs. 36.7 ± 8.5%, *p* = 0.014), and RV S′ velocity (8.0 ± 1.2 vs. 12.2 ± 2.9 cm/s, *p* < 0.001) compared to patients with RV FWS above median, while no difference was observed between the two groups in respect to TR V_max_ (3.0 ± 0.6 vs. 2.7 ± 0.6 m/s, *p* = 0.134). The estimated RV systolic pressures (RVSP) did not significantly differ between the groups (39.3 ± 16.6 vs. 35.1 ± 16.5 mmHg, *p* = 0.488).

**Figure 3:**
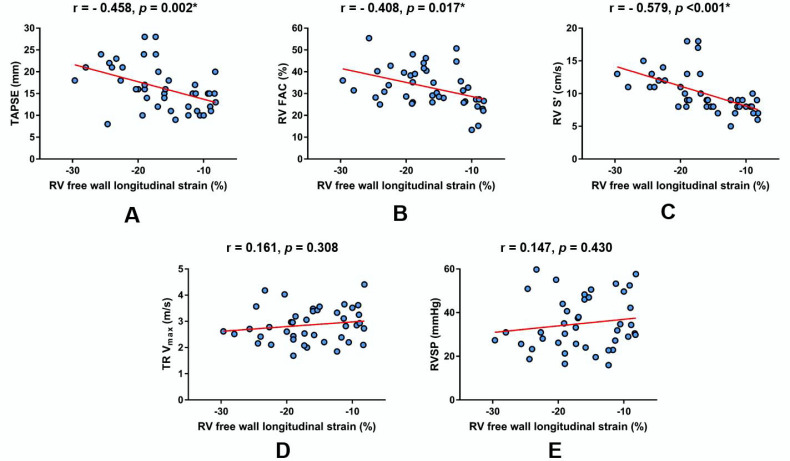
Linear regression plots showing the correlation of RV free wall longitudinal strain with respect to other echocardiographic parameters of RV function and estimated RV systolic pressure in the total patient cohort (*n* = 42). (**A**) TAPSE, (**B**) RV FAC, (**C**) RV S′, (**D**) TR Vmax, (**E**) RVSP. * Significant statistical association at two-tailed level. r-Pearson’s correlation coefficient. Abbreviations: RV FAC-right ventricular fractional area change; RV S′-myocardial systolic excursion velocity; RVSP-right ventricular systolic pressure (estimated); TAPSE-tricuspid annular plane systolic excursion; and TR V_max_-maximal tricuspid regurgitation velocity.

A bivariate Pearson correlation analysis showed that measured RV FWS values significantly correlated to TAPSE (Figure 3A), RV FAC (Figure 3B) and RV S′ (Figure 3C) but not to TR V_max_ (Figure 3D) and RVSP (Figure 3E), as shown below.

## 4. Discussion

In this study, we report that HF patients with more impaired RV function, as reflected in reduced RV free wall strain, had significantly higher circulating levels of total bilirubin and alkaline phosphatase, as well as prolonged PT-INR, reduced albumin, and decreased thrombocyte count, compared to patients that had better RV function (in the study defined as those above RW FWS median). Furthermore, RV FWS was independently associated with levels of total bilirubin and ALP reflecting a primarily cholestatic pattern of liver injury, while no significant association was observed with respect to AST and ALT as markers of hepatocellular injury. Similarly, no significant association was established between RV FWS and GGT. Importantly, a significant and independent association was established between RV FWS and markers of synthetic liver function such as albumin and PT-INR as well as thrombocyte count. Of conventional RV function echocardiography indices, RV FWS was significantly associated with TAPSE, RV FAC, and RV S′, while no significant association was established with TR V_max_ and estimated pulmonary artery pressures.

These are novel results in the context of RV mechanics and biochemical parameters of liver function since, to date, RV free wall strain as the specific marker of RV systolic function was not previously examined in the context of cardiohepatic interactions, particularly in the setting of HF. These results are more firmly established given the fact that no significant differences were found between compared groups in a vast majority of baseline parameters except for age, hospital length of stay, lymphocyte indices, and circulating NT-proBNP levels. Importantly, both groups were similar in respect to their baseline LV systolic function, LV global longitudinal strain, NYHA functional class, Fatty Liver Index (FLI), and renal function. A potential confounding effect of unrecognized PAH was also minimized since both groups had similar estimated pulmonary artery pressures and tricuspid regurgitation jet velocities. Even more, no significant differences were observed in pharmacotherapy use and circulating levels of CRP and relevant cardiac biomarkers reflecting cardiomyocyte injury, adverse ventricular remodeling, and sympathetic nervous system activity such as hs-cTnI, sST2, and catestatin. Finally, multiple linear regression analysis adjusted for relevant confounders validated these observations as RV FWS was independently associated with total bilirubin, ALP, albumin, thrombocyte count, and prolonged PT-INR but not to AST, ALT, and GGT.

Due to the inability to maintain adequate systemic perfusion of tissues and organ systems, HF must be perceived as a disease of all organs and all cells in the body. Arterial hypoperfusion dominates in the acute form of HF and leads to hypoxic hepatitis through a postulated mechanism of ischemic-reperfusion injury, while passive congestion is the major contributor in congestive hepatopathy secondary to chronic HF [29,30]. This passive congestion is the consequence of increased filling pressures and/or low cardiac output and hence, impaired hepatic perfusion leading to atrophy of hepatocytes in zone 3 and reduction in the ability of hepatocytes to extract oxygen [31,32]. Chronic hepatic stasis increases pressures within hepatic sinusoids thus precipitating bile duct damage by destroying endothelial cells and the interhepatocytic tight junctions that are the final frontier dividing extravascular space from the bile canaliculus [33]. Such congestive *milieu*, when chronically present, promotes atrophy of hepatocytes and causes perisinusoidal edema, thus significantly affecting diffusion of oxygen and nutrients to the hepatocytes [34]. Similarly, hepatic steatosis, a condition that is frequent in cardiac hepatopathy is promoted by comorbidities such as obesity, diabetes mellitus, and dyslipidemia, making the liver more susceptible to ischemia-reperfusion injury [35]. These comorbidities are all highly prevalent in chronic HF and have an established impact on prognosis in these patients [36], and our study was not exception given that nearly two-thirds of our cohort had dyslipidemia, one-third had diabetes, while exactly half of the patients were obese. However, both compared groups in our study had similar FLI, therefore, curtailing the possibility that fatty liver might drive observed differences.

In terms of laboratory derangements in cardiohepatic syndrome, it is recognized that passive liver congestion causes manifest elevations of hepatic and cholestatic enzymes, and this is linked to impaired hemodynamic status [31,37]. More specifically, acute cardiogenic liver injury is associated with significant elevations in aminotransferase levels, while congestive hepatopathy is associated with cholestatic profile of laboratory parameters and they correlate with disease severity and prognosis [38,39,40]. Furthermore, Poelzl and colleagues postulated that venous congestion rather than the forward failure has the strongest impact on the development of renal and hepatic derangements in chronic HF population [40]. Our findings seem to complement these observations since among patients with reduced RV FWS, we found that levels of total bilirubin and ALP were significantly higher when compared to patients with more preserved RV FWS, thus implicating that RV dysfunction (e.g., backward failure) is independently associated with abnormalities in biochemical parameters of liver function, as it was later validated in the regression analysis. More specifically, as in the study by Poelzl et al. conducted among chronic HF patients, we found that cholestatic enzymes, but not transaminases, were related to RV dysfunction, after adjustment for several clinical variables. Furthermore, we report that RV dysfunction was independently associated with markers reflecting synthetic liver function such as prolonged PT-INR and reduced albumin levels, while we also found significantly lower thrombocyte count among patients with a higher degree of RV dysfunction. Hypoalbuminemia in HF can serve as a biomarker of comorbidity burden, inflammation, malnutrition, and cachexia, while in patients with decompensated biventricular chronic HF, it was also attributed to splanchnic congestion [41,42]. 

RV dysfunction is highly prevalent and is an important harbinger of poor prognosis among patients with established left-sided HF, both in HFrEF and HFpEF clinical phenotypes [6,43]. In the study by Carluccio et al. conducted among HFrEF patients, it was demonstrated that RV FWS was superior to TAPSE for earlier detection of RV dysfunction and was able to prognosticate and reclassify even those patients that had normal TAPSE according to conventional guidelines [17]. Due to its complex anatomy and different response to hemodynamic challenges compared to the left ventricle, Carluccio et al. later demonstrated that RV FWS was prognostically superior to RV global longitudinal strain (GLS) among stable HFrEF patients due to its independence of interventricular septum (IVS) and minimization of the possible confounding effect of LV dysfunction since IVS is an integral part of the LV as well [18]. Notably, the predictive power of RV FWS was found independent of LVEF and LV GLS in the described population. In a study by Morris et al., RV FWS was able to detect subtle RV longitudinal systolic abnormalities in patients with HFrEF and, to a lesser extent, in HFpEF [44].

Taken together, RV FWS should be regarded as a reliable indicator of RV function among HFrEF patients, however, more evidence is required to establish its role in HFpEF patients. Finally, an important finding in our study is that RV FWS did not significantly correlate with RVSP and tricuspid regurgitation, while it did correlate with echocardiographic indices reflecting RV function of which strongest correlation was established with RV S′. Furthermore, both compared groups had similar LV systolic function and LV GLS. These findings suggest that we indeed can attribute observed results to isolated RV dysfunction, independently of potential confounding by concomitant LV dysfunction, elevated pulmonary pressures, and/or the degree of tricuspid regurgitation. Finally, all multiple linear regression models were also adjusted for baseline NYHA class reflecting functional syndrome severity, NT-proBNP levels, and NLR to account for these variables as potential confounders.

There are some limitations to this study. This was a single-center cross-sectional study. Therefore, no causal inferences can be made, and there is an inherent possibility of nonmeasured confounders. Furthermore, it could not be fully excluded that some of the subjects had relevant pulmonary hypertension that could affect RV function since no assessment with invasive right heart catheterization was performed. However, noninvasive assessment of pulmonary hypertension in patients with cardiac diseases with Doppler echocardiography provides good sensitivity (87%) and specificity (79%) and has a high degree of correlation (r = 0.87) with right heart catheterization [45]. Importantly, both groups that were compared had similar estimated pulmonary artery pressures and baseline LV function, and all measurements were made by the same expert echocardiographer, thus eliminating the potential interobserver variability. However, it should be noted that we did not measure a potential interobserver variability and reproducibility outcomes in this study since they were not prespecified in our study protocol, and this should be perceived as a limitation. Finally, a fairly modest sample size of our study might limit the generalizability of our results, however, RV FWS analysis is an advanced echocardiographic study that requires high expertise in speckle-tracking echocardiography and is time consuming in routine clinical practice. Also, other RV function indices along with the longitudinal deformation of LV were measured in each patient.

## 5. Conclusions

The results of this study, for the first time in the literature, show that reduced free wall strain of the RV is independently associated with adverse perturbations of biochemical parameters of liver function, particularly those reflecting cholestasis, among patients with acute worsening of HF. This relationship seems independent of baseline left ventricular systolic function, NYHA functional class, degree of tricuspid regurgitation, estimated pulmonary artery pressure, and natriuretic peptide levels. Furthermore, RV FWS was a good correlate of RV function concerning more conventional RV functional indices in the AWHF population. These findings might be of clinical value since reduced RV FWS, as an indicator of RV longitudinal systolic function, might indicate the presence of congestive hepatopathy among HF patients and vice versa—patients with a marked cholestatic pattern of liver injury per laboratory findings should be investigated for the possible RV dysfunction. 

## Figures and Tables

**Table 1:** Baseline characteristics of enrolled patient cohort according to the median of measured right ventricular free wall longitudinal strain (RV FWS).

Variable	Overall (*n* = 42)	Above Median (*n* = 21)	Below Median (*n* = 21)	*p*-Value *
Age, years	71.5 (IQR 62–76)	65 (IQR 54–73)	74 (IQR 68–77)	0.040
Female, %	50	57.1	42.9	0.355
Ischemic cardiomyopathy, %	45.2	42.9	47.6	0.757
NYHA class	3 (IQR 2–4)	3 (IQR 2–4)	3 (IQR 2–4)	0.351
Length of stay, days	14.1 ± 7.7	11.7 ± 7.0	16.6 ± 7.8	0.038
Fatty Liver Index	83 (IQR 67–91.3)	85 (IQR 61.5–95)	81 (IQR 71–89)	0.678
NAFLD, %	16.6	19.0	14.2	0.591
SBP, mmHg	140 (IQR 124–150)	140 (IQR 128–168)	140 (IQR 145–172)	0.215
LVEF, %	39.1 ± 16.0	40.2 ± 14.1	38.0 ± 18.0	0.650
LV GLS, %	−10.1 ± 5.5	−10.7 ± 4.6	−9.5 ± 6.3	0.489
eGFR, mL/min/1.73 m^2^	54.6 ± 26.4	56.2 ± 28.1	53.0 ± 25.3	0.700
BMI, kg/m^2^	30.5 ± 4.7	31.5 ± 5.5	29.5 ± 3.7	0.182
Beta-blocker, %	92.9	90.5	95.2	0.549
ACE-i or ARB, %	47.6	42.9	52.4	0.537
ARNi, %	31.0	23.8	38.1	0.317
MRA, %	40.5	38.1	42.9	0.753
Oral anticoagulant, %	45.2	42.9	47.6	0.757
Acetylsalicylic acid, %	38.1	42.9	33.3	0.525
Hemoglobin, g/dL	13.6 ± 2.1	13.3 ± 2.4	13.8 ± 1.8	0.416
APTT, s	27.4 ± 6.7	26.8 ± 8.6	27.9 ± 4.8	0.648
D-dimer, mg/L	1.5 ± 1.1	1.4 ± 1.4	1.6 ± 1.2	0.682
Lymphocytes, %	21.4 ± 8.2	23.8 ± 6.8	18.8 ± 8.9	0.047
NLR	4.6 ± 2.9	3.6 ± 1.9	5.7 ± 3.5	0.019
Sodium, mmol/L	139 (IQR 138–141)	139 (IQR 138–142)	139 (IQR 136–141)	0.232
Potassium, mmol/L	4.1 ± 0.5	4.1 ± 0.6	4.2 ± 0.4	0.539
Urea, mmol/L	9.4 (IQR 7.4–16.8)	8.3 (IQR 7.1–16.3)	11.3 (IQR 7.6–16.9)	0.285
Creatinine, mg/dL	1.21 (IQR 0.86–1.84)	1.06 (IQR 0.89–1.70)	1.33 (IQR 0.86–1.92)	0.308
Total cholesterol, mmol/L	4.2 ± 1.1	4.4 ± 1.1	3.9 ± 1.0	0.092
LDL cholesterol, mmol/L	2.5 ± 1.0	2.8 ± 1.1	2.3 ± 0.8	0.101
HDL cholesterol, mmol/L	1.2 ± 1.4	1.0 ± 0.3	1.3 ± 2.1	0.444
Triglycerides, mmol/L	1.7 ± 0.7	1.8 ± 0.7	1.5 ± 0.6	0.163
Fasting glucose, mmol/L	8 (IQR 6.3–10.8)	7.5 (IQR 6.1–10.3)	8.4 (IQR 6.7–12-4)	0.147
HbA1c, %	6.5 (IQR 6.1–7.6)	6.4 (IQR 6.1–6.9)	6.9 (IQR 6.1–8.3)	0.192
NT-proBNP, pg/mL	4413 (IQR 1552–12000)	2315 (IQR 1171–5590)	6266 (IQR 3089–12417)	0.038
sST2, ng/mL	29.9 (IQR 17.1–58.6)	29.7 (IQR 16–53)	30.0 (IQR 21.6–77)	0.458
Catestatin, ng/mL	6.2 (IQR 3.5–24)	8.6 (IQR 4.6–20.1)	6.2 (IQR 2.8–27.2)	0.513
hs-cTnI, ng/L	26.0 (IQR 14.2–72.5)	20.9 (IQR 10.2–30.5)	32.2 (IQR 17.9–130.3)	0.080
CRP, mg/L	8.9 (IQR 4.5–25.0)	7.4 (IQR 3.7–21.2)	9.9 (IQR 5.2–36.4)	0.235

Values are median (IQR-interquartile range), mean ± (SD-standard deviation) or percentage (%).* *p*-value for the independent samples *t*-test, Mann–Whitney U test, or Chi-square test, where appropriate. Abbreviations: ACE-i-angiotensin-converting enzyme inhibitor; ARB-angiotensin receptor blocker; ARNi-angiotensin receptor neprilysin inhibitor; BMI-body mass index; CRP-C-reactive protein; eGFR-estimated glomerular filtration rate; HbA1c-glycated hemoglobin A1c; HDL-high-density lipoprotein; hs-cTnI-high-sensitivity cardiac troponin I; LDL-low-density lipoprotein; LVEF-left ventricular ejection fraction; LV GLS-left ventricular global longitudinal strain; MRA-mineralocorticoid antagonist; NAFLD- nonalcoholic fatty liver disease; NLR-neutrophil-to-lymphocyte ratio; NT-proBNP-N-terminal pro-B-type natriuretic peptide; NYHA-New York Heart Association; sST2-soluble suppression of tumorigenicity 2; and SBP-systolic blood pressure.

**Table 2:** Values of laboratory parameters reflecting hepatic function and cholestasis according to the median of measured right ventricular free wall longitudinal strain (RV FWS).

Variable	Above Median (*n* = 21)	Below Median (*n* = 21)	*p*-Value *
AST, IU/L	26 (IQR 18–39)	30 (IQR 25–43)	0.147
ALT, IU/L	26 (IQR 16–46)	29 (IQR 19–50)	0.808
GGT, IU/L	30 (IQR 23–90)	47 (IQR 31–83)	0.134
Total bilirubin, µmol/L	12.9 ± 5.6	21.5 ± 8.9	<0.001
Alkaline phosphatase, IU/L	75 (IQR 60–87)	90 (IQR 72.5–122)	0.020
Thrombocyte count, × 10^9^/L	228 ± 53	187 ± 53	0.017
Albumin, g/L	39.1 ± 2.7	35.5 ± 4.8	0.005
Prothrombin time-INR	1.1 ± 0.23	1.7 ± 0.78	0.002

Values are median (interquartile range-IQR) or mean ± standard deviation. * *p*-value for the independent samples *t*-test or Mann–Whitney U test depending on the normality of distribution. Abbreviations: ALT-alanine aminotransferase; AST-aspartate aminotransferase; GGT-gamma-glutamyltransferase; and INR-international normalized ratio.
